# How traditional Norwegian outdoor activities are changing; a 10-year follow up in relation to sociodemographic factors

**DOI:** 10.3389/fspor.2024.1355776

**Published:** 2024-04-22

**Authors:** Thomas Birkedal Stenqvist, Elling Bere

**Affiliations:** ^1^ Department of Sport Science and Physical Education, University of Agder, Kristiansand, Norway; ^2^ Department of Health and Inequalities & Centre for Evaluation of Public Health Measures, Norwegian Institute of Public Health, Oslo, Norway

**Keywords:** outdoor activities, trends, gathering, hiking, growing, social inequality, friluftsliv

## Abstract

**Introduction:**

The study aims to evaluate the relationship between sociodemographic factors and changes in Norwegian outdoor activities between 2008 and 2018. Traditional outdoor activities, such as family trips in nature, the gathering of mushrooms and wild berries, and growing one's own plants to eat, are believed to have a positive impact on physical activity levels and health in general.

**Method:**

This study includes repeated cross-sectional surveys conducted in 38 randomly selected schools across two Norwegian counties. In 2008, 1,012 parents of 6th and 7th grade students from 27 schools completed a questionnaire. In 2018, 609 new parents from 25 schools participated. Variables were dichotomized. Descriptive analyses between groups were conducted using chi-square statistics. Binary logistic regression analyses were performed with the three outdoor activities as dependent variables, including year only (model 1), and then also gender, age (continuous), education (own and partners), and household income as independent variables (model 2).

**Results:**

Participation in weekly family trips in nature increased from 22% to 28% (*p* = 0.002), the OR for year 2018 vs. year 2008 was 1.51. Adjusted for sociodemographic factors, the OR remained stable and significant. Education was the only significant sociodemographic factor (OR = 1.60), indicating the odds of those with a higher education to be 60% higher to engage in weekly family trips in nature. Gathering of wild mushrooms and plants remained stable with time. Being female (OR = 1.44), age (OR = 1.049) and education (OR = 1.49) was related to gathering. An increase in growing plants to eat was observed with an increase from 42% to 51% (*p* < 0.001), OR = 1.33. However, it did not remain significant in model 2. Education was, in general, positively related to growing food (OR = 1.35).

**Conclusion:**

We observed a positive increase in family trips in nature over the period from 2008 to 2018. Furthermore, elder parents seem to be more involved in the long-rooted traditional Norwegian grow- and gather culture, and a social gradient is apparent as those with higher education do participate more often in traditional outdoor activities.

## 1 Introduction

Friluftsliv, plays a pivotal role in Scandinavian society, and especially in Norwegian culture and identity (1, 2). Friluftsliv refers to the practice of embracing open-air living and the deep connection with rural areas (3), and can be described as a form of non-motorized outdoor activity without formal competition, with simple means, with central viewpoints such as nature's depth, beauty, and greatness (4). Friluftsliv is often associated with traditional activities such as hiking, skiing, hunting, fishing, and gathering wild foods (3, 5). Furthermore, friluftsliv promotes health (6, 7). Activities such as hiking have been associated with positive subjective perceptions of physical health (8), and access to green spaces, in itself, is associated with increased health and well-being (9–12). In Norway, the proximity of nature to residential areas, even in major cities, allows for easy access and engagement with natural environments. Previous reports have indicated that a substantial proportion of the Norwegian population, up to 95%–98%, actively participates in some type of friluftsliv. However, traditional activities appear to be declining (13, 14), and that may negatively impact the health of the Norwegian population. Thus, a recent Government document from the Norwegian Ministry of Health and Care Services emphasizes the need for securing accessibility to green spaces for the Norwegian population (15). More advanced and expensive friluftsliv activities are on the rise, increasing inequality in the Norwegian society in relation to friluftsliv, despite the right to roam freely in Norwegian nature (14).

In recent years, there has been a growing scholarly interest in investigating the potential health benefits associated with exposure to natural vegetation and green spaces (15, 16). Moreover, the notion that spending time in nature can have positive effects on human health has been a longstanding belief (6, 17). Historically, humans had a hunter-gatherer lifestyle until the start of agriculture, during which the acquisition of food required significant physical activity (18). Consequently, engaging in movement and traversing natural landscapes in search of food resources have become deeply ingrained in human behavior. However, in modern industrialized societies, the availability of food has become more abundant, resulting in reduced physical effort required for sustenance (19). Nonetheless, wild foods gathered from natural environments are nutritionally dense and align with the principles of the New Nordic Diet, with Norway boasting a wide array of edible wild plants and mushrooms (20).

The investigation of cultivating edible foods within the context of friluftsliv has, however, received limited attention so far. Nevertheless, research have demonstrated positive associations between cultivating one's own food and the enhancement of health, happiness, and well-being (21, 22). Gardening has also been suggested as a behavior that both benefits individual and planetary health (23) and can be argued to be within the context of friluftsliv as described above.

A previous study examined the relationships between trips in nature, traditional methods for food procurement and weight status among parents of middle-school children (18). The findings indicated that individuals who engaged in nature trips and gathering activities had a lower prevalence of obesity. Upon controlling for sociodemographic factors, the authors noted that gathering behavior was influenced by sex and education level, with females and families of higher educational attainment engaging in gathering more frequently than those with lower education levels (18). These differences are interesting given that nature is free of charge and is, in theory, available for all Norwegians.

Building upon the findings, the primary objective of the present study was to examine the alterations in traditional outdoor activities, specifically family trips in nature, gathering, and cultivating one's own food, over a ten-year period from 2008 to 2018 in Norway. Additionally, the study aimed to investigate the associations between these activity patterns and various sociodemographic factors, including gender, age, educational attainment of both individuals and their partners, as well as household income, as these are factors that might influence participation in friluftsliv activities.

## 2 Methods

To address the aims, two repeated cross-sectional studies was performed. A questionnaire survey was conducted among sixth- and seventh-graders and one of their parents in 27 random schools in two Norwegian counties (Hedmark and Telemark) in 2008, as described in Bere and Westersjo (18) and again at the same schools in 2018. The questionnaire was initially part of a larger study by Bere, Hilsen and Klepp (24), and included questions regarding outdoor activities.

Family trips in nature were assessed with the question “How often does your family engage in trips in nature (forest or mountain area)?” The response alternatives were: Never; less than once a month; less than once a week; once a week; more than once a week. This item was dichotomized into ≥ once a week vs. less than once a week. Gathering of wild plants/mushrooms was assessed with the following statement: “I gather wild plants (e.g., berries) or mushrooms”, while growing was assessed with the statement: “I grow edible plants (e.g., berries and vegetables) at home for personal consumption”. These two items had three response alternatives: Yes often; Yes sometimes; No. They were dichoto­mized into ≥sometimes vs. never.

The parent respondent reported gender (male vs. female) and own age (years, continuous), own and partner's education level (as a measure of socio eco­nomic position, dichotomized into college/university education or not). A family education variable was created and categorized as high if both parents had higher education (one if single parent home) and low if not. Household income was an open question dichotomized at median at each survey.

Descriptive analyses of all variables from year 2008 and 2018 are presented in Table 1. Multivariate logistic regression analyses were then performed on the three different behaviors with time as the depend­ent variable (Table 2). Model 1 included time only. Model 2 included gender, age, family education level and income + Model 1. All analyses were conducted using Statistical Package for the Social Sciences (SPSS) for Windows (v. 29; IBM Corp., Armonk, NY, USA). and significance level was set as *p* < 0.05.

**Table 1:** Descriptive values of the included variables at 2008 and 2018.

	Year		*p*-value
	2008	2018	
Gender, parent (% female)	78	79	0.685
Age, parent (mean)	41.1	42.4	<0.001
Education, parent responding (% high)	54	69	<0.001
Education, family (% high)	37	49	<0.001
Household income (% high)	50	50	0.848
Family trips in nature (%, ≥once/week)	22	28	0.002
Gathering (%, ≥sometimes)	56	58	0.601
Growing (%, ≥sometimes)	42	51	<0.001

**Table 2:** Logistic regression showing odds-ratio (OR) of conducting traditional activities in relation to time, sex, age, family education and income.

		Model I		Model II	
		OR	CI (95%)	OR	CI (95%)
Family trips	Time (2018 vs. 2008)	1.51*	(1.15–1.98)	1.45*	(1.10–1.91)
	Gender (female vs. male)			1.02	(0.73–1.42)
	Age (years)			0.978	(0.951–1.005)
	Education, family (high vs. low)			1.60*	(1.21–2.12)
	Household income (high vs. low)			1.00	(0.76–1.33)
Gathering	Time (2018 vs. 2008)	1.10	(0.87–1.40)	0.98	(0.77–1.25)
	Gender (female vs. male)			1.44*	(1.10–1.91)
	Age (years)			1.046*	(1.021–1.071)
	Education, family (high vs. low)			1.49*	(1.17–1.91)
	Household income (high vs. low)			1.09	(0.86–1.39)
Growing	Time (2018 vs. 2008)	1.33*	(1.05–1.68)	1.25	(0.98–1.58)
	Gender (female vs. male)			1.02	(0.77–1.36)
	Age (years)			1.019	(0.996–1.043)
	Education, family (high vs. low)			1.36*	(1.07–1.72)
	Household income (high vs. low)			1.02	(0.80–1.29)

Model I, only containing time as the independent variable. Model II, contains model 1 + gender + age + family education and income.

* *p* < 0.05 CI; Confidence interval.

## 3 Results

In 2008, a total of 996 parents (of 1,712 eligible, 58%) of 6th and 7th graders derived from the 27 random schools participated in the survey. Of the 2008 study sample, 78% were women, 54% had higher education, and mean age was 41.1 years. In 2018, 609 new parents (of 1734 eligible, 35%) of 6th and 7th graders from 25 of 27 original schools participated. Of the 2018 study sample, 79% were women, 69% had higher education, and mean age was 42.4 years (Table 1). In 2008, 22% of families were engaged in family trips in nature at least once a week, which increased to 28% in 2018. Gathering of wild plants/mushrooms remained stable in the period of 2008–2018, 56% vs. 58% respectively. Gardening to grow plants for own consumption increased from 42% in 2008 to 51% in 2018 (Table 1).

The logistic regression revealed that family trips were 1.51 times more likely to happen in 2018 compared to 2008. Adjusted for sociodemographic factors, the likelihood remained stable. Family education was the only significant sociodemographic factor (OR = 1.60), indicating the odds of those with a higher family education to be 60% higher to engage in weekly family trips in nature. Gathering of wild plants and mushrooms remained stable from 2008 and did not change when adjusted for sociodemographic factors. Females were more likely to be engaged in gathering than males (OR = 1.44), older parents were more likely to engage in gathering (OR = 1.046) as well as those with higher family education (OR = 1.49) being more likely to engage in gathering. Growing plants for own consumption were 1.33 times more likely to happen in 2018 compared to 2008. When adjusting for sociodemographic factors, it did not remain significant. Only family education was positively related to growing plants (OR = 1.36).

## 4 Discussion

To the authors knowledge, only a few studies have investigated the evolution over time of traditional Norwegian outdoor activities in Norway (5, 13, 14). During the 10-year period investigated, family trips in nature were the only activity that increased, while gathering and growing remained unchanged. Furthermore, education was the only parameter which had significant influence on all three parameters, indicating that those with higher education were more engaged in all activities.

### 4.1 Family trips in nature

It has been reported that being out in nature in general is widely practiced and appreciated among Norwegians, and that yearly participation increased from 91% to 98% in the period of 1970–2004 (13, 14). Activities included multiple types such as bathing, walking, and cycling in nature alone or with friends/family. Another study showed a significant increase in forest walking from 55% in 1993 to 64% in 2013 (5). A recent survey on friluftsliv in Norway further highlights that walking in local areas has remained stable (∼80%–85%) with a peak of 94% in 2021, potentially due to the pandemic (14). These numbers likely differ due to methodological differences between studies, including activity classification, population, and frequency of trips (5, 13, 14). Despite their difference, the number of people involved in friluftsliv activities is still considered high. This is not surprising, as most Norwegians live in close proximity to natural areas (e.g., forest, seashore, or mountains), increasing the potential for engaging in local nature trips. Our findings show similar trends as described above, and it is positive that trips in nature are increasing due to their positive impact on public health (6, 7). Being engaged in a nature activities, or having greenspace available has been shown to be related to general subjective physical and mental health (8, 9, 16, 25, 26), and it is advised that outdoor activities are used to promote children's health (27, 28).

### 4.2 Gathering and growing

When Odden (13) investigated gathering (berry picking and mushrooms) as an outdoor activity, he found a decline in the age group of 35–54 years from 58% in 1970 to 41% in 2004. In a sub analysis, Odden (13) found that berry picking as a sole activity decreased, while mushroom gathering as a sole activity slightly increased. A recent report on friluftsliv in Norway, using the same data source as Odden, found that gathering further decreased to 30% in 2020 (14). In our population, we identified the prevalence of gathering to be 58% in 2018 yet it remained stable from 2008 to 2018. Gathering as an activity is beneficial, as it has been reported that the abundance of blueberries and cowberries alone may suffice to meet the national recommendations for fruit consumption in Norway (20). Hence, gathering can be seen as an activity that can positively influence diet quality as well, in addition to the positive effect of physical activity and being in nature.

Compared to the study of Odden (13) and Rafoss and Seippel (5), we also investigated growing of edible plants, an activity we argue is within the friluftsliv context as described in the introduction (3, 4). Growing plants for personal consumption was 1.33 times more likely to happen in 2018 compared to 2008 in the unadjusted analysis. However, in our adjusted model we did not observe an increase during the last 10 years of growing plants. In 2004 it was reported that time spent gardening had changed little over the last 30 years in Norway (29). In later years, there has been an increased focus on home gardening and urban agriculture for sustainable diets (30), including a Norwegian national strategy (31). Gardening has also been suggested as one important behavior for sustainable physical activity (23).

### 4.3 Socioeconomic status and friluftsliv activities

Norway is one of the richest and most egalitarian countries in the world, yet rather large inequalities are still observed in health and wellbeing (32). Education was the only variable that was significant for all three outcomes (family trips, gathering and growing) investigated. This is similar to previous reports (13, 14) identifying that people engaged in activities were predominantly those with higher education (92% in 2004 vs. 71%). Odden (13) also found a significant participation increase among those with higher education from 76% in 1970 to 92% in 2004 in trips in nature. Those participating in gathering were also higher (47%) among those with higher education compared to those with lower education (37%) in 2004. Odden, however, only identified a significant decrease from 1970 to 2004 among those with lower education, whereas higher education remained stable.

Household income did, however, not have any significant impact on the different activities. This is interesting and indicates that these activities in Norway are more influenced by cultural capital (i.e., education) (33) than economic capital (i.e., income). In affluent countries, with relatively few poor people, education is often more strongly related to health-related behaviors than income itself (34).

With the Norwegian “right to roam” the countryside (35), traditional activities are considered available to all, yet inequality still seems large and potentially increasing (14). Compared to more expensive friluftsliv activities such as alpine skiing and kayaking, the activities of family trips in nature, gathering and growing can be described as “free of charge” as nature and access to green spaces are readily available.

Even if the activity itself is free of charge, it might still be resource demanding, e.g., knowledge about where to and how to conduct the behavior, as well as knowledge about the importance of the activity (e.g., health effect). Also, there are differences between access to green space and nature between low and highly educated people, and access to e.g., second homes, often used as a base for friluftsliv activities (36).

In an effort to counteract the rising inequality of friluftsliv participation (14), the national concept of BUA was established in 2014. BUA (37) is a non-profit organization aiming to make it easier for people (especially children and young adults) to be engaged in various friluftsliv activities requiring specialized equipment by lending equipment out for a limited period free of charge. Frilager (38) is another regional non-profit organization that specializes in lending specialized equipment to schools and organizations, making it easier, cheaper, and more sustainable to teach friluftsliv. The impact on inequality of such ideal organizations is highly interesting and clearly needs further investigation.

### 4.4 Gender and age differences

Different from Rafoss and Seippel (5), who observed that females were more likely to be engaged in trips in nature, we did not observe such differences, similar to what Odden (13) found. In gathering, females were also more likely to be engaged in these types of activities. This is similar to that of Rafoss and Seippel (5) and Odden (13). This is not surprising, as gathering traditionally has been seen as a female activity, compared to more male-dominated activities such as hunting and fishing (5, 18).

Age also has an influence on both gathering and growing. In both cases, older parents were more likely to be engaged in these activities, and this is similar to the results of Odden (13) and Statistics Norway (14). Older parents were more likely to be engaged in gathering, which is similar to both Odden (13) and Rafoss and Seippel (5). This may be due to the fact that younger people are more engaged in modern activities such as mountaineering, skiing and mountain biking, thereby moving away from traditional activities (5).

### 4.5 Limitations

A limitation of the present study is that we only included parents from two of Norway's 19 counties, meaning the results are thus not necessarily generalizable to the adult population of Norway, as Norway is geographically diverse with both a varying landscape and varying socio-economic statuses. Furthermore, most of the participants (79%) were female, who may be more prone to gather and grow their own plants. A repeated cross-sectional study cannot draw any inferences about causality. Results might similarly have been influenced by a lower participation rate in 2018 than in 2008, further biasing the results. All measures were self-reported, and as such were always prone to bias, e.g., the “social-desirability bias” (39). These included answering the alternative ’sometimes’ for the ques­tions about gathering and growing, and might have had a different meaning for different people (18). Finally, only a few potential confounding factors were included in the present analysis (outdoor activities, sex, family education level and household income). Including other confounding factors (e.g., distance to nature and eating habits) might have altered the results. There are methodological differences between our study and both Odden (13) and Statistics Norway (14), including sample size, data collection methods, and classification/inclusions of activities. Furthermore, we collected data from a smaller region compared to both Odden (13) and Statistics Norway (14). Hence, the results should be interpreted with care, but they still add to an understanding of what influences participation in friluftsliv—a topic that needs to be further investigated.

## 5 Conclusion

Friluftsliv as a concept contains a vast variety of activities in free air. Our study highlighted that there were some changes in traditional friluftsliv activities, including that family trips in nature increased, while gathering and growing did not increase over a 10-year period. All activities were related to cultural capital and were conducted more often among highly educated people. Access to green spaces is considered beneficial, as outlined in both the Government documents, and research has linked green spaces to increased physical and mental health among both adults and children. It is therefore important that governments secure access to green spaces to promote health. Further research is needed to understand how socioeconomic status influences participation in friluftsliv. Measures taken such as BUA and Frilager are highly interesting, and more research is needed to investigate how these may impact social inequality. Furthermore, the Covid-19 pandemic may have altered how Norwegians use nature, and future research should try to address how this may have impacted friluftsliv.

## Data Availability

The raw data supporting the conclusions of this article will be made available by the authors, without undue reservation.
